# Lysosomal-Associated Transmembrane Protein 5 Promotes Proliferation, Migration, and Invasion of Clear Cell Renal Cell Carcinoma

**DOI:** 10.1155/2022/6334546

**Published:** 2022-11-02

**Authors:** Ruo-Hui Huang, Zi-Lu Ge, Gang Xu, Qing-Ming Zeng, Wei Xia, Guan-Cheng Xiao, Xiao-Feng Zou, Bin-Bin Zhang

**Affiliations:** ^1^Medical College of Soochow University, Suzhou, Jiangsu 215006, China; ^2^Department of Urology, First Affiliated Hospital of Gannan Medical University, Ganzhou, Jiang Xi 341000, China; ^3^Jiangxi Stone Prevention Engineering Technology Research Center, Ganzhou, Jiang Xi 341000, China; ^4^First Clinical Medical College, Gannan Medical University, Ganzhou, Jiang Xi 341000, China; ^5^Department of Cardiology, The First Affiliated Hospital of Zhengzhou University, No. 1 Jianshe East Road, Zhengzhou, Henan 450052, China

## Abstract

Clear cell renal cell carcinoma (ccRCC) is the most aggressive and deadly cancer of the urinary system and is regulated by multiple signaling pathways. However, the specific molecular mechanisms underlying ccRCC have not been fully studied or demonstrated. This study aimed to elucidate the function of lysosomal-associated transmembrane protein 5 (LAPTM5) in ccRCC cell lines and animal models and determine the potential underlying mechanisms. Our results demonstrated that LAPTM5 expression in patients with ccRCC was significantly higher in the tumor group than that in the adjacent nontumor group. Moreover, LAPTM5 promoted proliferation, migration, and invasion of ccRCC cells through the gain and loss of the function of LAPTM5 in 786-0 and Caki-1 cell lines. Similar results regarding LAPTM5 overexpression were obtained in BALB/c nude mice. In addition, LAPTM5 activated the Jun N-terminal kinase (JNK)/p38 signaling cascade by interacting with Ras-related C3 botulinum toxin substrate 1 (RAC1). Treatment with an RAC1 inhibitor eliminated the effects of LAPTM5 in ccRCC. In conclusion, these results indicate that LAPTM5 may be a new therapeutic target for ccRCC via activation of the RAC1-JNK/p38 axis.

## 1. Introduction

Renal cell carcinoma (RCC) is among the ten most common tumors in the world [1]. The incidence rate of RCC is third among all cancers worldwide, and its associated mortality rate ranks first [2]. Clear cell renal cell carcinoma (ccRCC) is the most common type of RCC, accounting for approximately 70% of all cases. Notably, the ccRCC incident rate demonstrates an increasing trend [3]. RCC treatment, including that for ccRCC, has transitioned from nonspecific immunotherapy to the targeted therapy and immunotherapeutic agents [3]. The development of novel treatment options have led to alleviated symptoms in some patients; however, the ccRCC mortality rate is high and the prognosis remains poor, particularly in patients with high pathological ccRCC grades and stages [4]. Thus, illustrating the underlying molecular mechanisms and identifying new therapeutic targets for ccRCC treatments is crucial.

Lysosomal-associated transmembrane protein 5 (LAPTM5), also known as E3 and CD40-ligand-activated specific transcript, is a member of the LAPTM family. LAPTM5 is transferred from the Golgi apparatus to lysosomes through interactions with NEDD4, an E3 ubiquitin-protein ligase [5–7]. In contrast to the two widely expressed family members LAPTM4a and 4b, LAPTM5 is primarily located in late endosomes and lysosomes [7]. LAPTM5 contains a ubiquitin interaction region, which interacts with E3 ubiquitin ligases and plays important roles in ubiquitination [7]. Functionally, LAPTM5 participates in the progression of cellular immunity, protein transport, apoptosis, and tumorigenesis [8–10]. A recent study showed that LAPTM5 may serve as a biomarker related to testicular germ cell tumor prognosis and diagnosis, and the survival rate was higher in the low LAPTM5 expression group, what's more, the author considered that LAPTM5 may affect the immune function [8]. In addition, previous studies suggest that LAPTM5 is related to the activation of mitogen-activated protein kinase (MAPK), NF-*κ*B, PI3K/Akt, and other pathways [8, 11]. These signaling pathways play key roles in the progression of numerous tumors, including ccRCC [12–14]. However, the role of LAPTM5 in ccRCC and its potential molecular mechanisms are yet to be fully elucidated.

Results obtained from The Cancer Genome Atlas (TCGA) database demonstrated that LAPTM5 expression levels were higher in ccRCC tissues than that in paracancerous tissues. Western blotting and reverse transcription quantitative (RT-q) polymerase chain reaction (PCR) revealed that LAPTM5 expression was higher in the human ccRCC tumor group than that in the corresponding nontumor group. Moreover, LAPTM5 overexpression promoted tumor proliferation, migration, and invasion in 786-0 and Caki-1cell lines, whereas LAPTM5 knockdown exerted the opposite effects. In addition, LAPTM5-overexpressed 786-0 cells were injected into nude mice and the same results were obtained. The weight, volume, and protein expression levels of migration-, invasion-, and proliferation-associated markers in LAPTM5-overexpressed mice were higher than those in the control group. Moreover, our results demonstrate that LAPTM5 activates the Jun N-terminal kinase (JNK)/p38 axis by binding to Ras-related C3 botulinum toxin substrate 1 (RAC1), and the effects of LAPTM5 on tumor growth were attenuated following treatment with an RAC1 inhibitor. In conclusion, the results of our study demonstrate that LAPTM5 regulates ccRCC development by regulating the RAC1-JNK/p38 signaling cascade.

## 2. Materials and Methods

### 2.1. Human ccRCC Samples

The present study included patients who were pathologically diagnosed with ccRCC postoperatively at the First Affiliated Hospital of Gannan Medical University. This study was approved by the Ethics Review Committee of the First Affiliated Hospital of Gannan Medical University, and informed consent was obtained from all patients prior to the treatment (approval no. CCSC-2021101201). This study was conducted in accordance with the principles of the Declaration of Helsinki.

### 2.2. Animal Models

All animal experiments were approved by the First Affiliated Hospital of Gannan Medical University and carried out in accordance with the guidelines for the care and use of experimental animals. The 786-0 cells stably transfected with LAPTM5 (2 × 10^6^ cells in 200 *μ*l phosphate buffered saline (PBS)) and the corresponding negative controls (200 *μ*l PBS) were injected subcutaneously into 6-week-old BALB/c nude mice (*n* = 6 per group). After 28 days, the mice were sacrificed by intraperitoneal injection of sodium pentobarbital (100 mg/kg), and death was verified by respiratory and cardiac arrest and pupil dilation. The masses and volumes of the obtained tumors were detected and subsequently photographed.

### 2.3. Cell Lines and Culture

Human 786-0 (cat. no. BNCC338472) and Caki-1 (cat. no. BNCC100682) cells were purchased from the Bena Culture Collection (Beijing, China). The 786-0 cells were cultured in RPMI-1640 medium (cat. no. 11875119; Gibco; Thermo Fisher Scientific, Inc., Waltham, MA, USA), 10% fetal bovine serum (FBS) (cat. no. 10099-141 Gibco; Thermo Fisher Scientific, Inc.), and 1% penicillin-streptomycin (PS). Caki-1 cells were cultured in Dulbecco's Modified Eagle Medium (DMEM) (cat. no. 11995040; Gibco; Thermo Fisher Scientific, Inc.), 10% FBS (cat. no. 10099-141 Gibco; Thermo Fisher Scientific, Inc.), and 1% PS. The cells were incubated at 37°C with 5% CO_2_. An RAC1 inhibitor, NSC 23766 (cat. no. S803101), was purchased from Selleck Chemicals (Houston, TX, USA).

### 2.4. Bioinformatics Analysis

RNA sequencing data were downloaded from TCGA database (https://portal.gdc.cancer.gov/). Differential gene analysis was performed using the *R* package “DESeq2” (*R* Foundation for Statistical Computing, Vienna, Austria). Moreover, the *R* package “ggplot2” was used to visualize the data. The “survminer” package was used to visualize the survival probability, and the survival package was used for statistical analysis of survival data. A *t*-test was used to analyze tumor stage correction between the LAPTM5 and control groups.

### 2.5. Plasmid Construction

For the LAPTM5-overexpressed plasmids, the full-length LAPTM5 cDNA was cloned into the lentiviral vector of pHAGE using cytomegalovirus promoter and FLAG antibodies. For LAPTM5 knockdown plasmids, three short hairpin (sh) RNAs were selected (Sigma–Aldrich, St. Louis, MO, USA; Merck KGaA, Darmstadt, Germany) for cloning into the lentiviral vector pLKO.1; expression was detected using Western blot and RT-qPCR. To determine the molecular mechanism, RAC1 was cloned into the pcDNA5 vector using HA antibody. All primers used in this study are listed in Table S1 in the Supplementary Material.

### 2.6. Cell Transfection

pMD2.G, psPAX2, and the corresponding recombinant plasmids (including the empty vector) were cotransfected into 293T cells, and the supernatant was collected after 24 and 48 h using polyethylenimine linear. The 786-0 and CAKI-1 cells transfected with supernatant and polybrene (10 *μ*l/ml) were added. After 48 h, puromycin (4 *μ*l/ml) was used to select and obtain target cells.

### 2.7. 5-Ethynyl-2′-Deoxyuridine Labeling Assay

Approximately 5,000 786-0 or Caki-1 cells were inoculated per well in 48-well plates and subsequently cultured in 5% CO_2_ at 37°C for 48 h. Then, 5-ethynyl-2′-deoxyuridine (EdU) reagent was added to the cell culture medium and incubated for 2 h. The plates were then fixed and stained according to the instructions of the EdU kit (cat. no. C0071S; Beyotime Institute of Biotechnology, Shanghai, China). Fluorescent images were captured to compare the proliferation of each group.

### 2.8. Colony Formation Assay

A total of 1,000–1,500 cells were inoculated per well into 6-well plates at 37°C and 5% CO_2_. Following 10–15 days of culture, the cells were fixed with 4% paraformaldehyde at room temperature for 20 min and subsequently stained with 0.1% crystal violet for 15 min prior to imaging.

### 2.9. Wound-Healing Assay

Multiple horizontal lines were drawn on the back of 6-well plates with a marker. A total of 5–10 × 10^5^ cells were inoculated into each well. After overnight incubation, the cells reached full confluence. Subsequently, a 20-*μ*l pipette tip was used to scratch along the previously marked line on the back of the plate. The cells were then treated with PBS 2–3 times and the medium was replaced with fresh DMEM with 1% FBS. Cells were cultured in a 37°C, 5% CO_2_ incubator and images were captured under the microscope after 0 and 24 h. ImageJ software (National Institutes of Health, Bethesda, MD, USA) was used to calculate wound healing in each group.

### 2.10. Transwell Assay

The migration and invasion ability of ccRCC cells were assessed using a transwell assay (cat. no. 3421; Corning Inc., Corning, NY, USA). The 786-0 cells were inoculated into the upper chamber of a 24-well plate. Following incubation at 37°C and 5% CO_2_ for 6 h, the migrating cells were fixed using 4% paraformaldehyde and stained with 0.1% crystal violet. The cells in the upper chamber were gently removed and imaged, and the number of migrating cells was counted. The upper chamber was coated with Matrigel and the cells were inoculated into the upper chamber. The aforementioned protocols were used for invasion. Caki-1 cells were incubated at 37°C and 5% CO_2_ for 24 h, and the aforementioned procedures were performed to determine the migration and invasion of Caki-1-1 cells.

### 2.11. RT-qPCR

Total RNA was extracted using 1 ml Trizol reagent (cat. no. 15596-026; Thermo Fisher Scientific), then total RNA was reverse transcribed into cDNA using a cDNA synthesis kit (cat. no. 04896866001; Roche Diagnostics GmbH, Basel, Switzerland). RT-qPCR was performed using SYBR green PCR master mix. Glyceraldehyde 3-phosphate dehydrogenase (GAPDH) was used as an internal control. All primer sequences are listed in Table S2 in the Supplementary Material.

### 2.12. Western Blot Analysis

Tissue blocks and cells were added to 1 ml lysate (radioimmunoprecipitation assay (RIPA), phenylmethylsulfonyl fluoride complete, PhosSTOP, NaF, and Na3VO4), and tissue blocks were crushed using a grinder (30 Hz/s for 90 sec). Both the cells and tissues were centrifuged at 4°C and 12,000 rpm for 30 min and the supernatant was collected. The protein concentration was determined using a bicinchoninic acid protein assay kit. For sample preparation, 10X dithiothreitol, 10X loading buffer, and RIPA buffer were added to the cells. The samples were subjected to 10% sodium dodecyl-sulfate polyacrylamide gel electrophoresis and subsequently transferred onto polyvinylidene difluoride membranes. Primary antibodies were added, and the membranes were incubated at 4°C overnight. Following the primary incubation, secondary antibodies were added for 1 h at room temperature (25°C). GAPDH was used as an internal control. The antibodies used in this study are listed in Table S3 in the Supplementary Material.

### 2.13. Coimmunoprecipitation Assay

LAPTM5-FLAG and RAC1-HA were constructed and cotransfected into 293T cells. After 24 h, samples were collected, and FLAG antibody was added and shaken at 4°C overnight. The subsequent immunoprecipitate was treated with precooled immunoprecipitation buffer 5–6 times and 2× loading buffer was added; the interaction between LAPTM5 and RAC1 was detected using HA and FLAG antibodies. The aforementioned protocols were repeated, and the target proteins were immunoprecipitated using the HA antibody; the interaction between LAPTM5 and RAC1 was detected using Western blot analysis.

### 2.14. Glutathione-S-Transferase Pulldown Assay

GST-HA-LAPTM5, FLAG-LAPTM5, GST-HA-RAC1, and FLAG-RAC1 were overexpressed and lysed using lysis buffer (cocktail of 50 mM Na_2_HPO^4^, 300 mM NaCl, and 1% TritonX-100). GST-HA-LAPTM5 proteins were purified using glutathione-S-transferase (GST) beads, and GST-HA-LAPTM5 and FLAG-RAC1 were mixed and incubated overnight at 4°C. The beads were treated with buffer (20 mM; 150 mM NaCl and 0.2% Triton X-100) three times, resuspended in 2× loading buffer, and analyzed using Western blotting. GST-HA-RAC1 and FLAG-LAPTM5 were overexpressed, and the aforementioned protocol was repeated.

### 2.15. Statistical Analysis

All data are presented as means± standard deviations and were statistically analyzed using SPSS software (version 23.0; IBM Corp., Armonk, NY, USA). For normally distributed data, a two-tailed Student's *t*-test was used for comparisons between the two groups; one-way analysis of variance was used for comparisons among multiple groups, followed by Bonferroni's *post hoc* analysis (data meeting the homogeneity of variance) or Tamhane's T2 analysis (missing variance). For data with skewed distributions, nonparametric statistical analysis was performed using the Mann–Whitney *U* test for the two groups and Kruskal–Wallis test for multiple groups. P < 0.05 was considered statistically significant.

## 3. Results

### 3.1. LAPTM5 is Highly Expressed and Related to Poor Prognosis in ccRCC

**Figure 1:**
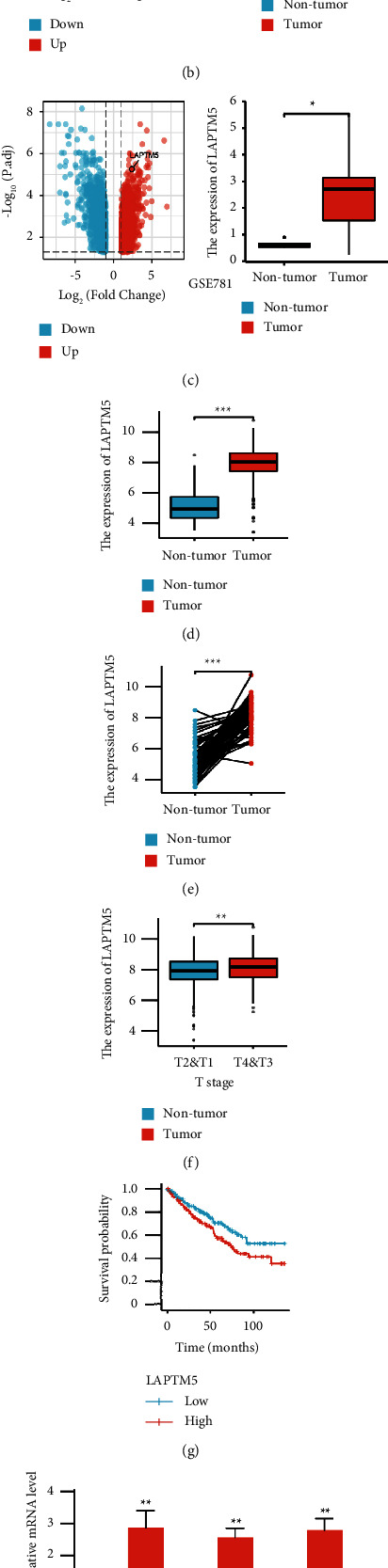
LAPTM5 expression is increased in patients with ccRCC and is associated with the stage and prognosis of ccRCC. (a) LAPTM5 is differentially expressed in numerous tumors, including ccRCC (statistical description of tumors are listed in Table S4 in the Supplementary Material). (b) and (c)Expression of LAPTM5 in tumor tissues and healthy tissues (GSE 6344 and GSE 781). (d) Expression of LAPTM5 in tumor tissues of patients with ccRCC and adjacent healthy tissues (tumor, *n* = 539; nontumor, *n* = 72) in TCGA. (e) Expression of LAPTM5 in paired tumor tissues and adjacent healthy tissues in patients with ccRCC (*n* = 72) in TCGA. (f) Expression of LAPTM5 in different TNM stages (T1 and T2, *n* = 349; T3 and T4, *n* = 190) in TCGA. (g) Overall survival curve of ccRCC with high and low LAPTM5 expression levels (high, top 30%, *n* = 179; low, bottom 30%, *n* = 178) in TCGA. (h) mRNA expression of LAPTM5 in ccRCC tissues and adjacent healthy tissues (*n* = 3 independent experiments), basic information of patients are listed in Table S5 in the Supplementary Material. (i) Western blot analysis of LAPTM5 in every group (*n* = 3 independent experiments). (j) Western blot analysis of LAPTM5 in every group (*n* = 3 independent experiments). *∗* fnlowast p < 0.05 vs. nontumor or low, *∗∗* fnlowast *∗*p < 0.01 vs. nontumor or low, *∗∗∗* fnlowast *∗∗*p < 0.001 vs. nontumor or low. NS, not significant; abbreviation: LAPTM5, lysosomal-associated transmembrane protein 5; ccRCC, clear cell renal clear cell carcinoma; TCGA, The Cancer Genome Atlas; T2&T1: TNM stages II and I; T4&T3: TNM stages IV and III; Case 1–3: patients with ccRCC 1–3; T1–T3: tumor tissue of patient 1–3; N1–N3: nontumor tissue (adjacent tissue) of patient 1–3.

To explore the role of LAPTM5 in ccRCC, the LAPTM5 expression levels were determined in tumor and adjacent healthy tissues of patients with ccRCC obtained from TCGA database and GEO database (GSE 6344 and GSE 781). The results suggested that LAPTM5 expression was higher in patients with ccRCC than that of patients in the control group (Figures 1(a)–1(e)). Moreover, the results of TCGA database showed that LAPTM5 is significantly associated with tumor stage. The LAPTM5 expression levels in stages III and IV were significantly higher than those in stages I and II (Figure 1(f)). Survival analysis was subsequently conducted, and the results suggest that increased LAPTM5 expression is closely related to poor prognosis of ccRCC (Figure 1(g)). To further verify these results, RT-qPCR and Western blot analyses were performed in human ccRCC tissues. The results obtained from the RT-qPCR and Western blot analyses support the previously obtained results (Figures 1(h)–1(i)). We selected 786-0, Caki-1, and 769-P cell lines to explore the LAPTM5 expression in ccRCC cell lines. Compared with the renal epithelial cell lines HK-2, the results showed that LAPTM5 was upregulated in three ccRCC cell lines (Figure 1(j)) and we chose 786-0 and Caki-1 for the further experiment. In conclusion, LAPTM5 may be involved in ccRCC progression.

### 3.2. LAPTM5 Promotes ccRCC Proliferation *In Vitro*

**Figure 2:**
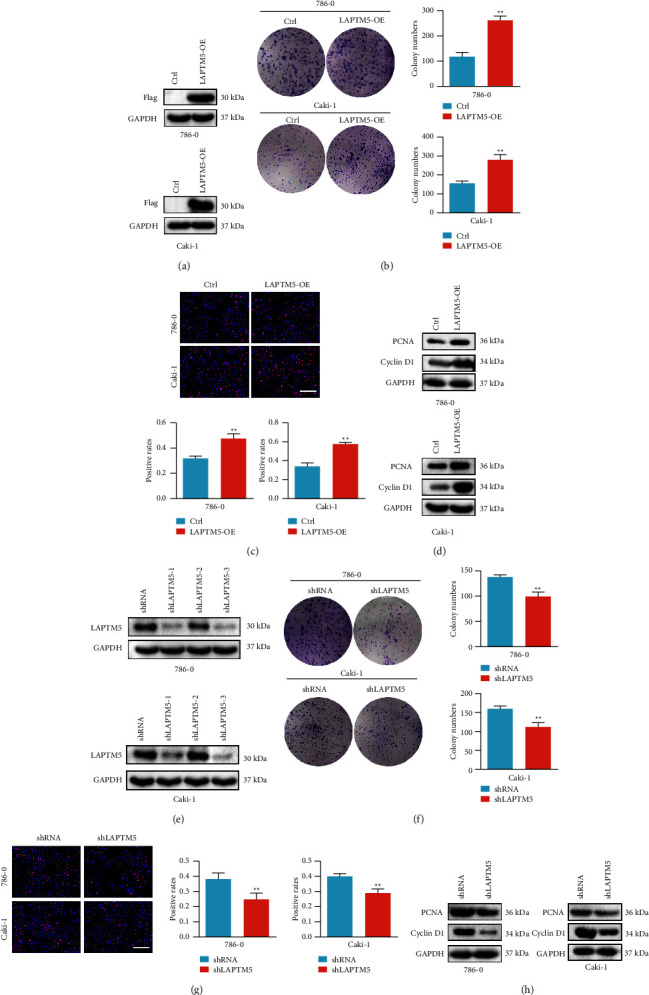
LAPTM5 promotes ccRCC proliferation *in vitro*. (a) Western blot analysis of LAPTM5 expression in 786-0 and Caki-1 cells using a FLAG antibody. (b) Colony formation of the LAPTM5-overexpression or control groups in 786-0 and CAKI-1 cell lines. (c) EdU assay results of the LAPTM5-overexpression group or control group. Scale bar, 200 *μ*m. (d) The expression of proliferation molecular markers. (e) Western blot analysis of LAPTM5 expression in 786-0 and Caki-1 using the LAPTM5 antibody. (f) Colony formation of the shLAPTM5 and shRNA groups. (g) EdU assay results of the shLAPTM5 or shRNA groups. Scale bar, 200 *μ*m. (h) Western blotting results of proliferation markers in shLAPTM5 and shRNA groups. *∗∗*P < 0.01 vs. control or shRNA group. Abbreviations: LAPTM5, lysosomal-associated transmembrane protein 5; ccRCC, clear cell renal clear cell carcinoma; LAPTM5-OE: LAPTM5 overexpression; shLAPTM5: short-hairpin LAPTM5; shRNA, short-hairpin RNA; PCNA: proliferating cell nuclear antigen.

LAPTM5 overexpression (Figure 2(a)) and knockdown were performed in 786-0 and Caki-1 cell lines. Following LAPTM5 knockdown using three different plasmids, shLAPTM5-3 was selected for follow-up experiments (Figure 2(e)). The effects of LAPTM5 on cell proliferation were determined using a clone formation assay, EdU staining, and proliferation marker levels. We found that the number of cell clones and the cell proliferation rate (Figures 2(b) and 2(c)) in the LAPTM5-overexpression group were higher than that in controls, indicating that LAPTM5 promoted cell proliferation in ccRCC. In addition, the expression of the molecular markers of proliferation, namely, proliferating cell nuclear antigen (PCNA) and cyclin-D1, were determined in both cell lines, suggesting that both markers were higher in the LAPTM5-overexpression group, compared with that in the control group (Figure 2(d)). Moreover, the above results showed that LAPTM5 knockdown decreased the number of cell clones, cell proliferation rate, and molecular marker expression (PCNA and cyclin-D1) in 786-0 and Caki-1 cells (Figures 2(f)–2(h)). Thus, these results demonstrate that LAPTM5 promotes cell proliferation *in vitro*.

### 3.3. LAPTM5 Promotes Migration and Invasion of ccRCC *In Vitro*

**Figure 3:**
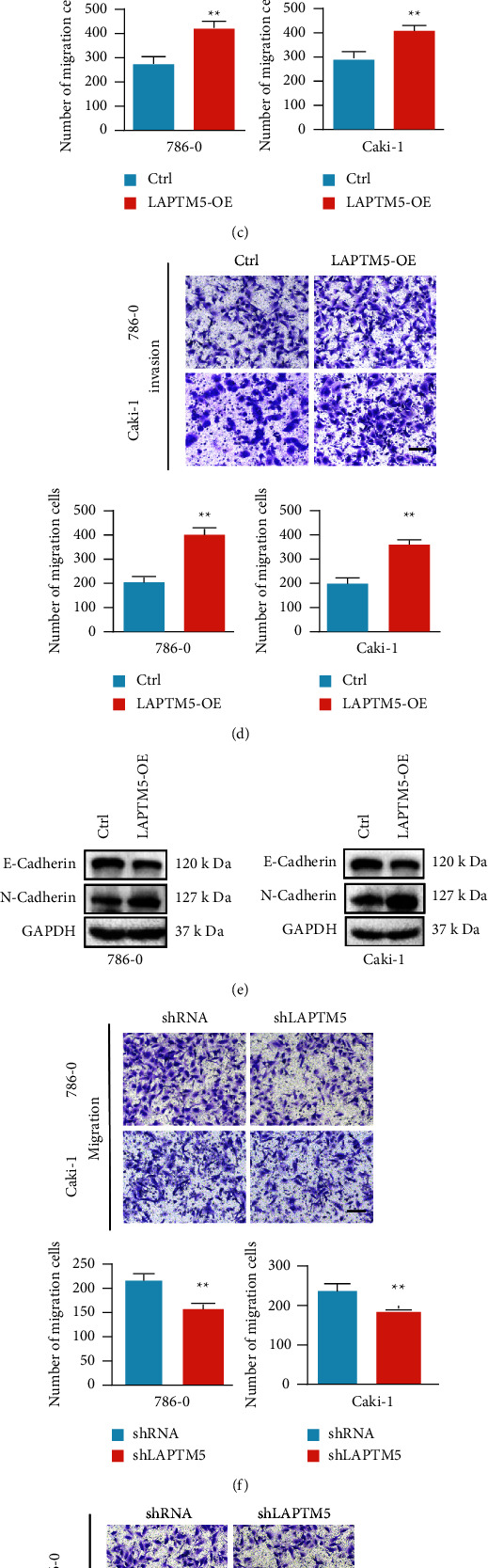
LAPTM5 promotes ccRCC cell migration and invasion *in vitro*. (a) Results of the wound-healing assay of the LAPTM5-overexpression or control groups in both 786-0 and Caki-1 cells. (b) Results of the wound-healing assay of the shLAPTM5 or shRNA groups in 786-0 and Caki-1 cells. (c) Migration of 786-0/Caki-1 LAPTM5-overexpression or 786-0/Caki-1 control groups. Scale bar, 100 *μ*m. (d) Invasion of 786-0/Caki-1 LAPTM5-overexpression or 786-0/Caki-1 control groups. Scale bar, 100 *μ*m. (e) Protein expression of migration- and invasion-associated molecular makers in 786-0/Caki-1 LAPTM5-overexpression or 786-0/Caki-1 control groups. (f)–(h) Migration, invasion, and expression of migration- and invasion-associated markers in 786-0/Caki-1 shLAPTM5 or 786-0/Caki-1 shRNA groups. Scale bar, 100 *μ*m. *∗∗*p < 0.01 vs. control or shRNA group. Abbreviations: LAPTM5, lysosomal-associated transmembrane protein 5; ccRCC, clear cell renal cell carcinoma; LAPTM5-OE: LAPTM5 overexpression; shLAPTM5: short-hairpin LAPTM5; shRNA, short-hairpin RNA.

To verify the role of LAPTM5 in 786-0 cell migration, a wound healing assay was performed, which suggested that overexpressed LAPTM5 promoted cell migration, whereas knockdown inhibited cell migration (Figure 3(a)). These results were validated in Caki-1 cells (Figure 3(b)). The function of LAPMT5 in cell invasion and migration was determined using a transwell assay, which revealed that LAPTM5 overexpression promoted 786-0 and Caki-1 cell migration (Figure 3(c)) and invasion (Figure 3(d)). The expression of migration- and invasion-associated molecular markers was also detected. The results demonstrated that N-cadherin expression was increased and E-cadherin expression was decreased in the LAPTM5-overexpression group (Figure 3(e)). In addition, the transwell assay revealed that LAPTM5 knockdown inhibited 786-0 and Caki-1 cell migration and invasion (Figures 3(f) and 3(g)). Moreover, the N-cadherin level was reduced and E-cadherin was promoted in the LAPTM5-knockdown group compared with that in the control group (Figure 3(h)). In conclusion, these findings suggest that LAPTM5 promotes the migration and invasion of ccRCC cells *in vitro*.

### 3.4. LAPTM5 Promotes ccRCC Development and Growth *In Vivo*

**Figure 4:**
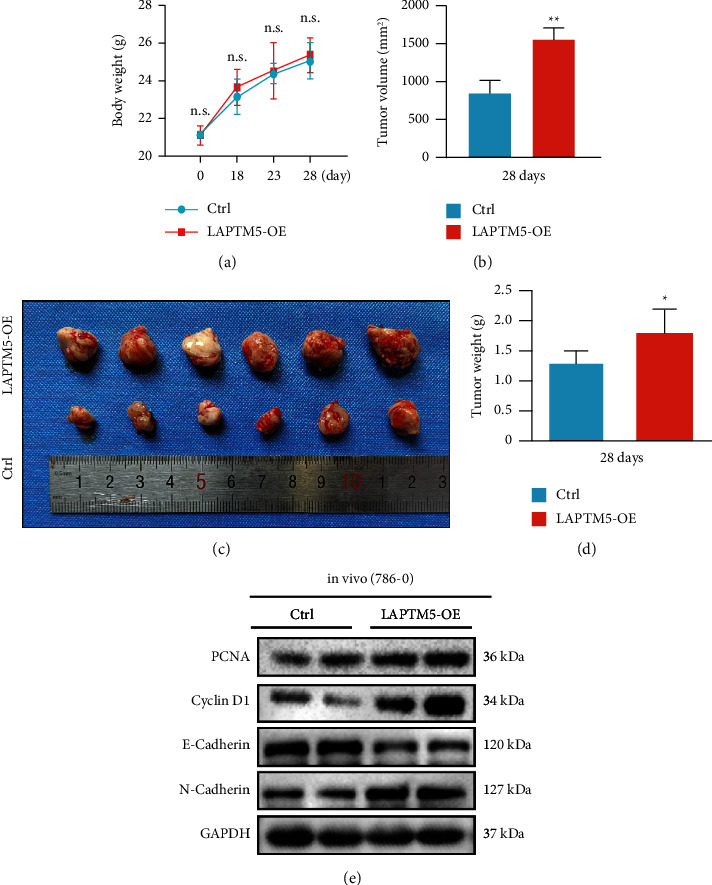
LAPTM5 promotes ccRCC growth *in vivo*. (a) Body weight of mice at 0, 18, 23, and 28 days after injecting 786-0 cells (*n* = 6 per group). (b) Tumor volume of LAPTM5-overexpression mice or control mice at day 28 (*n* = 6 per group). (c) Mass of the tumor in LAPTM5-overexpression or control groups (*n* = 6 per group). (d) Images of 786-0-LAPTM5-overexpression injected mice and 786-0-control injected mice (*n* = 6). (e) Protein expression of proliferation-, migration-, and invasion-associated markers in 786-0-LAPTM5-overexpression injected mice and 786-0-control injected mice (*n* = 3 independent experiments). ^*∗*^P < 0.05, ^*∗∗*^P < 0.01 vs. control group. Abbreviations: LAPTM5, lysosomal-associated transmembrane protein 5; LAPTM5-OE: LAPTM5 overexpression.

The effects of LAPTM5 were evaluated *in vivo* using subcutaneous tumorigenesis experiments in BALB/c mice. The 786-0 cell line with LAPTM5 overexpression was injected into mice, and the time until tumor visualization was observed. Notably, the tumor could be visualized after 18 days. Weight changes in the BALB/c mice were also observed from days 18 to 28, and the tumor volume was recorded on day 28. No significant changes in the weight of the mice were observed (Figure 4(a)), and the tumor volume in the LAMPT5-overexpression group was larger than that in the control group (Figure 4(b)). In addition, the tumor mass in the LAPTM5 overexpression group was significantly larger than that in the control group (Figures 4(c) and 4(d)). Molecular markers of proliferation, migration, and invasion were detected in ccRCC mice using Western blotting. The results demonstrated that PCNA, cyclin-D1, and N-cadherin levels increased, while E-cadherin levels decreased (Figure 4(e)). Overall, these results indicate that LAPTM5 promotes tumor growth *in vivo.*

### 3.5. LAPTM5 Activates JNK/p38 Pathway by Binding to RAC1

**Figure 5:**
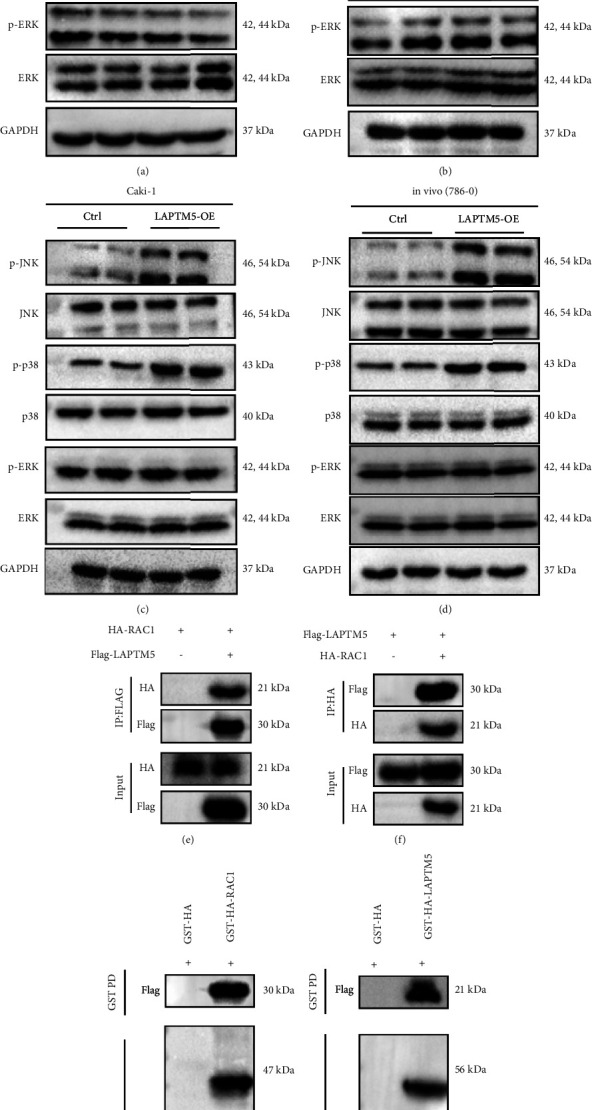
LAPTM5 promotes activation of the JNK/p38 axis. (a) Protein expression of phosphorylated and total MAPK in the 786-0-LAPTM5-overexpression and 786-0-control groups. (b) Protein expression of phosphorylated and total MAPK in 786-0-shLAPTM5 and 786-0-shRNA groups. (c) Protein expression of phosphorylated and total MAPK in the Caki-1-LAPTM5-overexpression and Caki-1-control groups. (d) Protein expression of phosphorylated and total MAPK in 786-0-LAPTM5-overexpression injected mice and 786-0-control injected mice. (e) and (f) Interaction between LAPTM5 and RAC1 in 293T cells. (g) and (h) Interaction between LAPTM5 and RAC1 *in vitro*. ^*∗*^P < 0.05, ^*∗∗*^P < 0.01 vs. control or shRNA groups. n = 3 independent experiments. Abbreviations: LAPTM5, lysosomal-associated transmembrane protein 5; shRNA, short-hairpin RNA; RAC1, Ras-related C3 botulinum toxin substrate 1; FLAG-LAPTM5: LAPTM5 and FLAG fusion protein; HA-LAPTM5: LAPTM5 and HA fusion protein; FLAG-RAC1: RAC1 and FLAG fusion protein; HA-RAC1: RAC1 and HA fusion protein.

The potential mechanisms underlying ccRCC were explored in the present study. Notably, the MAPK pathway is widely involved in numerous cellular activities, such as proliferation, migration, and apoptosis and is closely associated with ccRCC. The results demonstrated that LAPTM5 overexpression activated p38 and JNK phosphorylation, whereas the expression levels of total and phosphorylated extracellular signal-regulated kinase (ERK) did not change (Figure 5(a)). LAPTM5 knockdown inhibited p38 and JNK phosphorylation in 786-0 cells, although no change was observed in ERK expression levels (Figure 5(b)). We also found that LAPTM5 overexpressed activated the phosphorylation of JNK and p8 in Caki-1 cell lines (Figure 5(c)). In addition, results consistent with those previously described were obtained *in vivo* following subcutaneous tumorigenesis in BALB/c nude mice (Figure 5(d)). Thus, these results suggest that LAPTM5 regulates ccRCC growth through MAPK pathways. Moreover, our results demonstrate that RAC1 upstream from MAPK interacts with LAPTM5 (Figures 5(e) and 5(f)). We also conducted GST-pulldown assays to verify the direct interaction between LAPTM5 and RAC1 (Figure 5(g) and 5(h)). Thus, LAPTM5 might regulate MAPK activation by interacting with RAC1.

### 3.6. RAC1 Knockdown Regulates ccRCC Phenotype through LAPTM5

**Figure 6:**
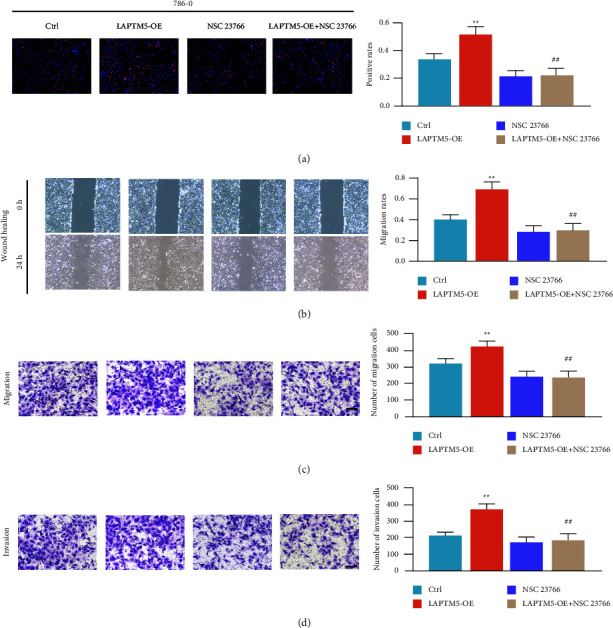
RAC1 inhibition is a key step in regulating the phenotype of ccRCC. (a) EdU assay images of the LAPTM5-overexpression, control, RAC1 inhibitor, and LAPTM5-overexpression + RAC1 inhibitor groups in 786-0 cells. Scale bar, 200 *μ*m. (b) Wound-healing results of the control, LAPTM5-overexpression, RAC1 inhibitor, and LAPTM5-overexpression + RAC1 inhibitor groups in 786-0 cells. (c) and (d) Migration and invasion of the control, LAPTM5-overexpression, RAC1 inhibitor, and LAPTM5-overexpression + RAC1 inhibitor groups in 786-0 cells. Scale bar, 100 *μ*m. ^*∗∗*^P < 0.01 vs. control, ##fn##P < 0.01 vs. Abbreviations: LAPTM5-OE: LAPTM5-overexpression. RAC1, Ras-related C3 botulinum toxin substrate 1; ccRCC, clear cell renal clear cell carcinoma; LAPTM5, lysosomal-associated transmembrane protein 5.

Treatment with an RAC1 inhibitor (NSC 23766) was administered to determine whether RAC1 is crucial to LAPTM5 for growth regulation in ccRCC. The wound healing and EdU assays demonstrate that the promoting effects of LAPTM5 on tumor proliferation were significantly weakened following RAC1 inhibitor treatment (Figures 6(a) and 6(b)). Moreover, the effects of LAPTM5 on tumor migration and invasion were eliminated following RAC1 inhibitor treatment (Figures 6(c) and 6(d)). These findings support the notion that LAPTM5 regulates ccRCC growth through the RAC1 signaling pathway.

## 4. Discussion

ccRCC poses a severe threat to the lives of patients and involves numerous signaling pathways. Exploring and clarifying the potential mechanisms of the pathways involved in ccRCC is crucial for the development of novel ccRCC treatment options. We focused on the function of LAPTM5 as a marker of ccRCC, and the results demonstrate that LAPTM5 regulates JNK and p38 phosphorylation by directly interacting with RAC1 to promote proliferation, migration, and invasion of ccRCC. When RAC1 is inhibited, the ccRCC phenotype is no longer affected by LAPTM5. Based on our results, we suggest that LAPTM5 can serve as an oncogene in the development of ccRCC.

As a lysosomal transmembrane protein, LAPTM5 is widely involved in various cell functions, including protein transport, immune cell activation, signal pathway activation, autophagy, and oxidative stress [9, 15–20]. LAPTM5 is also widely involved in the progression of various cellular activities and signaling pathways. LAPTM5 plays a negative regulatory role in T and B cell activation [21] and is widely involved in tumor growth. In addition, LAPTM5 inhibits tumor invasion, clonogenicity, and tumorigenicity by inhibiting NF-*κ*B activation, which may act as a therapeutic target for CD40+gliomas [22]. LAPTM5 knockdown leads to apoptosis in lung cancer cells; thus, inhibiting LAPTM5 expression has potential for the treatment of cancer [23]. LAPTM5 also mediates cell death in neuroblastoma. Notably, the E3 ubiquitin ligase, ITCH, promotes degradation by ubiquitination to prevent LAPTM5-mediated programmed death [24]. In bladder cancer, LAPTM5 knockdown inhibited cell proliferation and the cell cycle [25]. ccRCC is a urinary system tumor, and the potential function of LAPTM5 in ccRCC remains to be fully explored and elucidated.

A previous study found that LAPTM5 was downregulated in most human tumors, including RCC. Notably, the authors compared 15 RCC cell lines with nontumor tissues and found that the mRNA expression of LAPTM5 was downregulated[10]. This was an extensive screening through RT-PCR. However, in our study, we found that LAPTM5 was upregulated in ccRCC tumor tissues compared with that in the adjacent healthy tissues using Western blot and RT-PCR, which is consistent with previous bioinformatics analyses of kidney renal clear cell carcinoma (Figure 1(a)). Proliferation, migration, and invasion are key factors in tumor development [26, 27]. In our study, we found that LAPTM5 promoted proliferation, migration, and invasion of ccRCC cells *in vitro* and promoted tumor growth and survival *in vivo*; however, the underlying mechanisms remain unknown. Numerous pathways are involved in the progression of ccRCC, including MAPK, wingless/integrated, and PI3K/Akt pathways [28–30]. JNK, p38, and ERK are important components of the MAPK pathway [31]. In LAPTM5 knockdown macrophages, the activation of tumor necrosis factor receptor-mediated MAPK and NF-*κ*B pathways was reduced, indicating that LAPTM5 is a positive regulator of the inflammatory signaling axis in macrophages [21]. In addition, LAPTM5 regulates protein stability. Notably, A20 is a powerful inhibitor of the NF-*κ*B and MAPK signaling cascade, and LAPTM5 directly promotes A20 degradation by binding with it in HeLa cells in the endolysosomal system [21]. Thus, we hypothesized that LAPTM5 regulates ccRCC via MAPK pathways. Our findings further demonstrate that LAPTM5 promotes p38 and JNK phosphorylation in ccRCC cells and in animal models by directly binding to RAC1. Numerous previous studies have reported that p38 and JNK are involved in ccRCC survival and growth and play a significant regulatory role in ccRCC tumorigenesis, metastasis, and angiogenesis [32–34], which is consistent with our findings. Notably, ERK is a key downstream factor of MAPK and is involved in ccRCC progression [35, 36]. However, no significant changes in ERK expression were observed in the present study. ERK may be regulated by other important molecules, and further investigations are therefore required.

RAC1 belongs to the Rho guanosine triphosphatase family and acts as a “molecular switch” in various cell signal transduction processes. In addition, RAC1 is an upstream molecule of MAPK located in lysosomes [37]. When RAC1 is activated, it plays a crucial role in tumorigenesis, growth, and death [38–40]. In gliomas, high RAC1 expression promotes tumor migration and invasion by inducing epithelial-mesenchymal transition and increasing matrix metalloproteinase expression [41]. Zhu *et al.* demonstrated that GINS complex subunit 4 promotes gastric cancer development by regulating RAC1 [39]. RAC1 is overexpressed in ccRCC cells, thereby promoting proliferation, migration, metastasis, and angiogenesis and is required for MAPK pathway activation [42]. In the present study, we hypothesized that LAPTM5 regulates the MAPK pathway by interacting with RAC1. The results of our study demonstrate an interaction between LAPTM5 and RAC1. Moreover, these results demonstrate that treatment with an RAC1 inhibitor rescued the ccRCC phenotype caused by LAPTM5. Collectively, these findings indicate that LAPTM5 regulates ccRCC growth by activating the RAC1-JNK/p38 axis.

This study had some limitations. Notably, LAPTM5 knockdown was not performed *in vivo*, and the interaction between LAPTM5, RAC1, and MAPK requires further investigation.

## 5. Conclusions

In conclusion, the function and molecular mechanisms of LAPMT5 were investigated in ccRCC. Our results demonstrate that LAPTM5 regulates ccRCC cell proliferation, migration, and invasion by interacting with RAC1 and activating downstream JNK/p38 pathways.

## Data Availability

The datasets used and/or analyzed during the current study are available from the corresponding author on reasonable request.

## Abbreviations

ccRCC: Clear cell renal cell carcinoma
EdU: 5-ethynyl-2′-deoxyuridine
ERK: extracellular signal-regulated kinase
GAPDH: Glyceraldehyde 3-phosphate dehydrogenase
GST: Glutathione-S-transferase
JNK: Jun N-terminal kinase
LAPTM5: Lysosomal-associated transmembrane protein 5
MAPK: Mitogen-activated protein kinase
PCR: Polymerase chain reaction
PCNA: Proliferating cell nuclear antigen
RAC1: Ras-related C3 botulinum toxin substrate1
RCC: Renal cell carcinoma
RT-q: Reverse transcription quantitative
sh: Short hairpin.

## Supplementary Materials

Supplementary Materials Table S1: the primers for plasmid construction. Table S2: the primers for RT-PCR. Table S3: the primary antibodies. Table S4: statistical description of tumors mentioned in the text from TGCA. Table S5: the basic information of patients in this study.
