# Supplementary material for: Lysosomal-Associated Transmembrane Protein 5 Promotes Proliferation, Migration, and Invasion of Clear Cell Renal Cell Carcinoma
Source: J Oncol. 2022 Nov 2;2022:6334546. doi: 10.1155/2022/6334546 (PMC9646302; doi:10.1155/2022/6334546)
Supplement: Supplementary Materials — Table S1: the primers for plasmid construction. Table S2: the primers for RT-PCR. Table S3: the primary antibodies. Table S4: statistical description of tumors mentioned in the text from TGCA. Table S5: the basic information of patients in this study. [file 6334546.f1.docx]

**Supplementary Data**

LAPTM5 promotes the proliferation, migration and invasion of renal clear cell carcinoma

Running title: HUANG *et al*: LAPTM5 REGULATES THE PROGRESSION OF RENAL CLEAR CELL CARCINOMA

Ruo-Hui Huang^1,2,3*^, Zi-Lu Ge^4*^, Gang Xu^2,3^, Qing-Ming Zeng^2,3^, Wei Xia^2,3^, Guan-Cheng Xiao^2,3^, Xiao-Feng Zou^2,3#^ and Bin-Bin Zhang ^5#^

^1^Medical College of Soochow University, Suzhou, Jiangsu, 215006, China

^2^Department of Urology, First Affiliated Hospital of Gannan Medical University, Gan Zhou, Jiang Xi, 341000, China

^3^ Jiangxi Stone Prevention Engineering Technology Research Center, Gan Zhou, Jiang Xi, 341000, China

^4^First Clinical Medical College, Gannan Medical University, Ganzhou, Jiangxi, 341000, China

^5^Department of Cardiology, The First Affiliated Hospital of Zhengzhou University, No.1 Jianshe East Road, Zhengzhou, 450052, China

*These authors contributed equally to this work

Corresponding authors: Bin-Bin Zhang, Tel: 86-0371-67967622; Fax: 86-0371-67967626; E-mail: zbazhx@163.com; Xiao-Feng Zou, Tel: 0797-8689138; E-mail: [gyfyurology@yeah.net](mailto:gyfyurology@yeah.net).

**Key words:** Renal clear cell carcinoma, LAPTM5, RAC1, tumor growth

**Table S1. The primers for plasmid construction.**

| Gene name | Forward primer (human) | Reverse primer (human) |
| --- | --- | --- |
| LAPTM5 | TCGGGTTTAAACGGATCCatggacccccgcttgtc | GGGCCCTCTAGACTCGAGcacctctgagtatgggggtgg |
| RAC1 | TCGGGTTTAAACGGATCCatgcaggccatcaagtgtgtg | GGGCCCTCTAGACTCGAGttacaacagcaggcattttctcttcct |
| shLAPTM5-1 | CCGGCACAACTGGGTCAACGCTTTACTCG  AGTAAAGCGTTGACCCAGTTGTGTTTTTG | AATTCAAAAACACAACTGGGTCAACGCTTT  ACTCGAGTAAAGCGTTGACCCAGTTGTG |
| shLAPTM5-2 | CCGGCCTACTGATCGGCGTAGTCAACTCG  AGTTGACTACGCCGATCAGTAGGTTTTTG | AATTCAAAAACCTACTGATCGGCGTAGTC  AACTCGAGTTGACTACGCCGATCAGTAGG |
| shLAPTM5-3 | CCGGATGTGATCATGAGCGTCTTGTCTCG  AGACAAGACGCTCATGATCACATTTTTTG | AATTCAAAAAATGTGATCATGAGCGTCTT  GTCTCGAGACAAGACGCTCATGATCACAT |

**Table S2. The primers for RT-PCR.**

| Gene name | Forward primer (human) | Reverse primer (human) |
| --- | --- | --- |
| *Laptm5* | GCTACCTCAGGATCGCTGAC | ATTTGCAGGGACAGGAAGGG |
| *Gapdh* | ACTCCACTCACGGCAAATTC | TCTCCATGGTGGTGAAGACA |

**Table S3. The primary antibodies.**

| Anbibody | Manufacturer | Catalogue number |
| --- | --- | --- |
| LAPTM5 | Proteintech | AG30809 |
| p-ERK | CST | 4370 |
| ERK | CST | 4695 |
| p-JNK | CST | 4668 |
| JNK | CST | 9252 |
| p-p38 | CST | 4511 |
| p38 | CST | 9212 |
| PCNA | Proteintech | 10205-2-AP |
| Cyclin-D1 | Proteintech | 26939-1-AP |
| E-Cadherin | ABclonal | A11492 |
| N-Cadherin | ABclonal | A3045 |
| GAPDH | CST | 2118 |

**Table S4. Statistical description of tumors mentioned in the text from TGCA.**

| Group1 | Group2 | Number | Min | Max | Median | IQR | Mean | SD | SE |
| --- | --- | --- | --- | --- | --- | --- | --- | --- | --- |
| ACC | Normal | 128 | 0 | 7.313 | 5.57 | 0.912 | 5.453 | 0.856 | 0.076 |
| ACC | Tumor | 77 | 2.195 | 8.478 | 4.417 | 1.552 | 4.712 | 1.336 | 0.152 |
| BLCA | Normal | 28 | 4.286 | 7.751 | 5.568 | 0.989 | 5.672 | 0.882 | 0.167 |
| BLCA | Tumor | 407 | 0.807 | 9.475 | 5.768 | 2.272 | 5.587 | 1.587 | 0.079 |
| BRCA | Normal | 292 | 3.124 | 8.274 | 5.811 | 1.008 | 5.844 | 0.768 | 0.045 |
| BRCA | Tumor | 1099 | 3.313 | 9.645 | 6.811 | 1.246 | 6.756 | 0.939 | 0.028 |
| CESC | Normal | 13 | 3.275 | 6.158 | 4.84 | 1.34 | 4.686 | 0.89 | 0.247 |
| CESC | Tumor | 306 | 2.348 | 8.745 | 5.882 | 1.524 | 5.787 | 1.158 | 0.066 |
| CHOL | Normal | 9 | 3.046 | 5.545 | 4.485 | 0.619 | 4.335 | 0.753 | 0.251 |
| CHOL | Tumor | 36 | 3.662 | 9.166 | 5.99 | 1.335 | 5.947 | 1.163 | 0.194 |
| COAD | Normal | 349 | 0 | 8.56 | 5.052 | 1.396 | 5.031 | 1.104 | 0.059 |
| COAD | Tumor | 290 | 2.064 | 8.581 | 5.57 | 1.957 | 5.469 | 1.309 | 0.077 |
| DLBC | Normal | 444 | 4.594 | 11.482 | 9.395 | 1.287 | 9.203 | 1.005 | 0.048 |
| DLBC | Tumor | 47 | 7.354 | 10.579 | 9.536 | 0.695 | 9.499 | 0.643 | 0.094 |
| ESCA | Normal | 666 | 0 | 7.676 | 4.459 | 1.149 | 4.456 | 0.976 | 0.038 |
| ESCA | Tumor | 182 | 2.759 | 8.8 | 5.841 | 1.688 | 5.689 | 1.199 | 0.089 |
| GBM | Normal | 1157 | 0 | 9.49 | 3.425 | 1.98 | 3.594 | 1.496 | 0.044 |
| GBM | Tumor | 166 | 3.99 | 10.261 | 8.023 | 1.279 | 7.876 | 1.032 | 0.08 |
| HNSC | Normal | 44 | 2.952 | 8.001 | 4.91 | 1.181 | 5.016 | 0.932 | 0.14 |
| HNSC | Tumor | 520 | 2.266 | 8.771 | 5.932 | 1.747 | 5.907 | 1.263 | 0.055 |
| KICH | Normal | 53 | 0 | 7.635 | 4.396 | 1.645 | 4.229 | 1.475 | 0.203 |
| KICH | Tumor | 66 | 1.48 | 8.703 | 4.635 | 1.701 | 4.661 | 1.319 | 0.162 |
| KIRC | Normal | 100 | 0 | 7.828 | 4.361 | 1.492 | 4.43 | 1.321 | 0.132 |
| KIRC | Tumor | 531 | 2.683 | 10.049 | 7.306 | 1.163 | 7.217 | 0.986 | 0.043 |
| KIRP | Normal | 60 | 0 | 7.635 | 4.066 | 1.14 | 4.18 | 1.404 | 0.181 |
| KIRP | Tumor | 289 | 1.233 | 9.408 | 6.33 | 1.65 | 6.291 | 1.246 | 0.073 |
| LAML | Normal | 70 | 6.682 | 7.074 | 6.872 | 0.086 | 6.881 | 0.084 | 0.01 |
| LAML | Tumor | 173 | 6.298 | 10.763 | 9.276 | 0.874 | 9.167 | 0.811 | 0.062 |
| LGG | Normal | 1152 | 0 | 9.49 | 3.399 | 1.979 | 3.584 | 1.491 | 0.044 |
| LGG | Tumor | 523 | 2.614 | 9.79 | 6.647 | 1.691 | 6.665 | 1.144 | 0.05 |
| LIHC | Normal | 160 | 1.411 | 7.593 | 3.877 | 1.837 | 3.877 | 1.219 | 0.096 |
| LIHC | Tumor | 371 | 1.705 | 8.857 | 4.882 | 1.674 | 4.904 | 1.177 | 0.061 |
| LUAD | Normal | 347 | 0 | 9.816 | 8.025 | 0.806 | 7.935 | 0.772 | 0.041 |
| LUAD | Tumor | 515 | 3.034 | 9.555 | 7.275 | 1.219 | 7.135 | 1.035 | 0.046 |
| LUSC | Normal | 338 | 0 | 9.536 | 8.001 | 0.82 | 7.921 | 0.771 | 0.042 |
| LUSC | Tumor | 498 | 2.223 | 9.415 | 6.649 | 1.55 | 6.533 | 1.19 | 0.053 |
| MESO | Tumor | 87 | 5.529 | 9.212 | 7.254 | 1.131 | 7.26 | 0.874 | 0.094 |
| OV | Normal | 88 | 0.956 | 7.056 | 3.578 | 1.517 | 3.525 | 1.268 | 0.135 |
| OV | Tumor | 427 | 0 | 9.333 | 6.281 | 2.023 | 6.095 | 1.45 | 0.07 |
| PAAD | Normal | 171 | 0 | 9.134 | 2.852 | 1.337 | 3.082 | 1.344 | 0.103 |
| PAAD | Tumor | 179 | 2.897 | 9.488 | 6.972 | 1.413 | 6.835 | 1.17 | 0.087 |
| PCPG | Normal | 3 | 5.009 | 6.002 | 5.523 | 0.496 | 5.511 | 0.496 | 0.286 |
| PCPG | Tumor | 182 | 1.86 | 8.999 | 4.934 | 1.557 | 4.97 | 1.085 | 0.08 |
| PRAD | Normal | 152 | 1.816 | 7.514 | 4.854 | 1.199 | 4.889 | 0.894 | 0.072 |
| PRAD | Tumor | 496 | 2.336 | 7.974 | 4.896 | 1.189 | 4.927 | 0.91 | 0.041 |
| READ | Normal | 318 | 0 | 8.56 | 4.99 | 1.273 | 4.909 | 1.073 | 0.06 |
| READ | Tumor | 93 | 2.524 | 8.022 | 5.302 | 1.34 | 5.393 | 1.166 | 0.121 |
| SARC | Normal | 2 | 4.402 | 5.128 | 4.765 | 0.363 | 4.765 | 0.513 | 0.363 |
| SARC | Tumor | 262 | 2.269 | 9.998 | 6.798 | 2.239 | 6.641 | 1.621 | 0.1 |
| SKCM | Normal | 813 | 0.151 | 8.577 | 3.686 | 3.278 | 3.12 | 1.7 | 0.06 |
| SKCM | Tumor | 469 | 0.766 | 9.845 | 6.235 | 2.175 | 6.244 | 1.515 | 0.07 |
| STAD | Normal | 210 | 0 | 7.76 | 4.427 | 1.559 | 4.461 | 1.15 | 0.079 |
| STAD | Tumor | 414 | 2.862 | 10.357 | 6.499 | 1.738 | 6.418 | 1.219 | 0.06 |
| TGCT | Normal | 165 | 0.824 | 6.19 | 2.43 | 1.146 | 2.583 | 1.011 | 0.079 |
| TGCT | Tumor | 154 | 2.651 | 9.123 | 6.221 | 1.438 | 6.25 | 1.146 | 0.092 |
| THCA | Normal | 338 | 0 | 8.984 | 5.174 | 1.086 | 5.293 | 1.009 | 0.055 |
| THCA | Tumor | 512 | 2.516 | 8.525 | 5.609 | 1.45 | 5.58 | 1.116 | 0.049 |
| THYM | Normal | 446 | 4.594 | 11.482 | 9.388 | 1.293 | 9.197 | 1.007 | 0.048 |
| THYM | Tumor | 119 | 4.572 | 8.892 | 7.287 | 0.931 | 7.235 | 0.692 | 0.063 |
| UCEC | Normal | 101 | 2.506 | 6.497 | 4.545 | 1.134 | 4.547 | 0.807 | 0.08 |
| UCEC | Tumor | 181 | 2.797 | 7.947 | 5.602 | 2.002 | 5.555 | 1.203 | 0.089 |
| UCS | Normal | 78 | 2.506 | 6.074 | 4.41 | 1.185 | 4.411 | 0.776 | 0.088 |
| UCS | Tumor | 57 | 2.705 | 7.469 | 5.223 | 1.664 | 5.358 | 1.102 | 0.146 |
| UVM | Tumor | 79 | 1.778 | 7.561 | 3.599 | 2.102 | 3.948 | 1.462 | 0.164 |

**Table S5. The basic information of patients in this study.**

| ID | Sex | Age | BMI | Tumor location | TNM stage | RENAL score | Size |
| --- | --- | --- | --- | --- | --- | --- | --- |
| 1 | Female | 45 | 22 | Right | T2bN0M0 | 7 | 7*8*10.5cm |
| 2 | Male | 28 | 24 | Left | T1bN0M0 | 6 | 5*4*6cm |
| 3 | Male | 67 | 20 | Right | T2aN0M0 | 8 | 6*7*8cm |

Abbreviations: BMI: Body Mass Index; TNM: tumor node metastasis.
